# Management of Traumatic Bone Cyst in a 3-Year-Old Child: A Rare Case Report

**DOI:** 10.5005/jp-journals-10005-1169

**Published:** 2012-12-05

**Authors:** Naveen Reddy Banda, Ullal Anand Nayak, Kambalimath Halaswamy Vishwanath, Divya S Sharma, Vishal Khandelwal

**Affiliations:** Reader, Department of Pedodontics and Preventive Dentistry, Modern Dental College and Research Centre, Indore, Madhya Pradesh, India e-mail: drreddybanda@gmail.com; Professor and Head, Department of Pedodontics and Preventive Dentistry, Mahatma Gandhi Dental College, Jaipur, Rajasthan, India; Professor, Department of Pedodontics and Preventive Dentistry, Modern Dental College and Research Centre, Indore, Madhya Pradesh, India; Professor, Department of Pedodontics and Preventive Dentistry, Modern Dental College and Research Centre, Indore, Madhya Pradesh, India; Senior Lecturer, Department of Pedodontics and Preventive Dentistry Modern Dental College and Research Centre, Indore, Madhya Pradesh India

**Keywords:** Traumatic bone cyst, Trauma, Acrylic splint

## Abstract

The following case report describes a case of traumatic bone cyst (TBC) with classical clinical features occurring as a rare combination in a very young female patient with a traumatic etiology and its management using acrylic splint postsurgery.

**How to cite this article:** Banda NR, Nayak UA, Vishwanath KH, Sharma DS, Khandelwal V. Management of Traumatic Bone Cyst in a 3-Year-Old Child: A Rare Case Report. Int J Clin Pediatr Dent 2012;5(3):213-216.

## INTRODUCTION

Traumatic bone cyst (TBC) is known by a great many names in the literature, such as hemorrhagic bone cyst, simple bone cyst, solitary bone cyst, idiopathic bone cyst, primary bone cyst, blood cyst or unicameral bone cyst.^1^ It is an uncommon lesion, first recognized by Virchow in 1876.^2^ The WHO classification describes TBC as a nonneoplastic osseous lesion. It is defined as ‘an intraosseous cyst having a tenuous lining of connective tissue with no epithelium’. It has an unclear etiology and course and the most accepted explanation being hemorrhage theory, which states that trauma leads to intramedullary hemorrhage and subsequently formed blood clot liquefies destroying the adjacent bone by enzymatic activity.^1,3^

TBC in jaws is mainly diagnosed in young patients most frequently during the second decade of life, and very rarely seen affecting children <5 years. The gender distribution is reported to be quite even, but some have reported male predilection with 3:2 ratio.^1,4^

Most TBC’s are located in the mandibular body between canine and third molar, followed by mandibular symphysis in the maxillofacial region (Incisor region—as in young persons, this area contains hematopoietic marrow).^5^

TBC is usually asymptomatic, other unusual complaints include pain, tooth sensitivity, paresthesia, delayed eruption of permanent teeth, displacement or root resorption and rarely pathologic fractures of the mandible.^6^

On radiological examination, TBC usually appears as unilocular, well-defined radiolucency, with characteristic ‘scalloping effect’ extending between the roots of the teeth and sparing of cortical boundary of the crypt around developing tooth. TBC have tendency to grow along long axis of bone causing minimal expansion, and rarely inferior cortex expansion or thinning is produced by larger lesions. Inferior alveolar canal appear to be displaced inferiorly or its cortical outline may be resorbed completely.^7,8^

The definitive diagnosis of TBC is invariably achieved at surgery when an empty bone cavity without epithelial lining is observed, leaving very little except normal bone and occasional fibrous tissues curetted from the cavity wall for histopathologists.^3,7,9^

The widely recommended treatment for TBC is surgical exploration followed by curettage of bony walls, which may provoke bleeding in the cavity, and hemorrhage forms clot in the cavity which is eventually replaced by bone, and enhancing the healing process. It is believed that in some cases, especially younger patients there may be a spontaneous resolution.^1,3,9,10^

After surgical exploration with or without curettage, obliteration of the defect by new bone formation is generally rapid. Even large defects may show normal radiographic findings within 3 to 12 months after exploration.^3,10^ The prognosis is excellent and recurrence is unusual. Periodic follow-up and radiographic examinations are advisable until complete resolution has been confirmed.^11,12^

The following case report describes a case of TBC with classical clinical features with a rare combination of a very young female patient with a traumatic etiology and its management.

## CASE REPORT

**Fig. 1:**
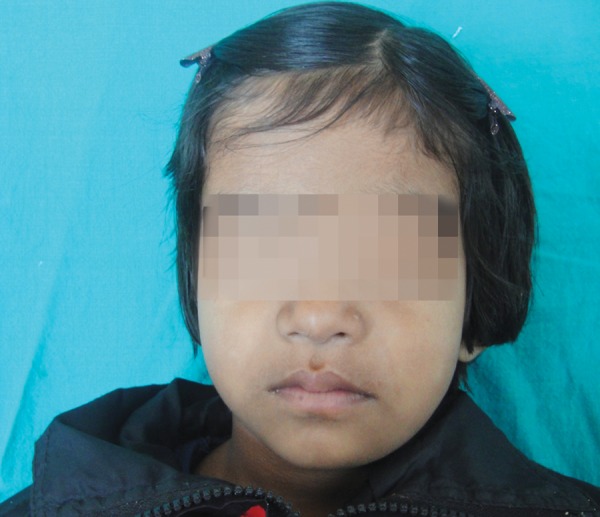
Extraoral swelling of left lower third of the face

**Fig. 2:**
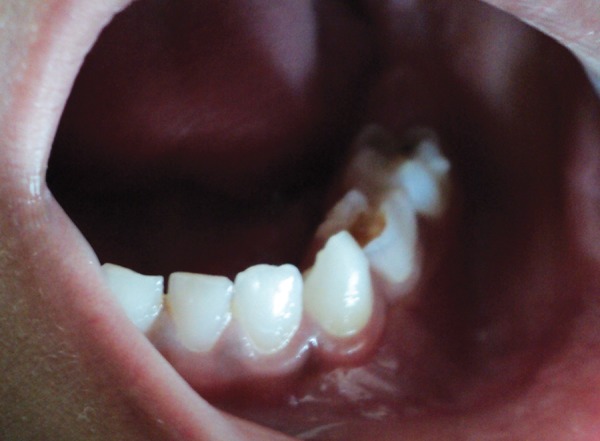
Intraoral swelling present in the deciduous canine, molar region

**Fig. 3:**
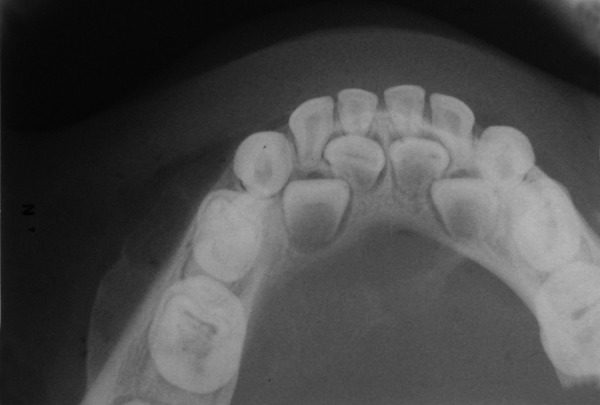
Occlusal radiograph showing well-defined radiolucency crossing midline with thinning of the buccal and lingual cortical plates

**Fig. 4:**
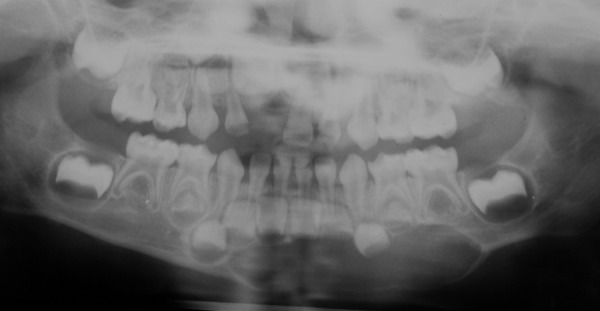
Panoramic radiograph showing a partly well-defined, solitary unilocular radiolucency with scalloped and corticated borders

**Fig. 5:**
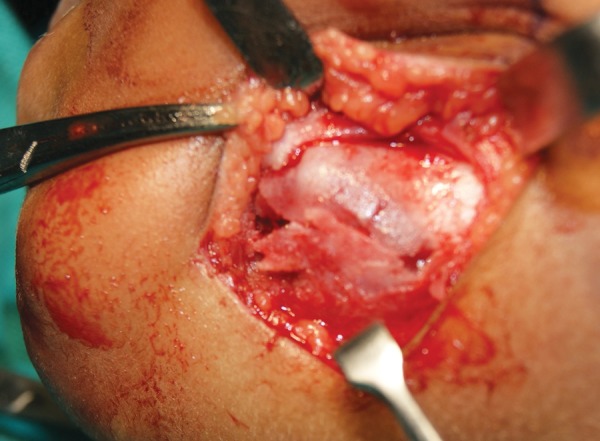
Extraoral photograph of the surgical site

**Fig. 6:**
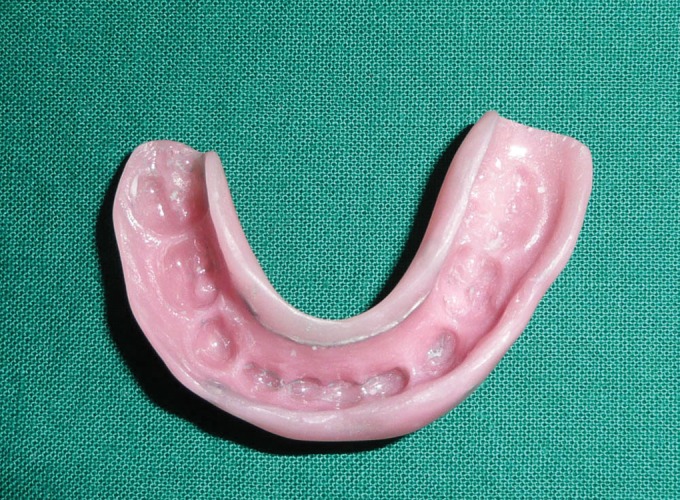
Fabricated occlusal splint

**Fig. 7:**
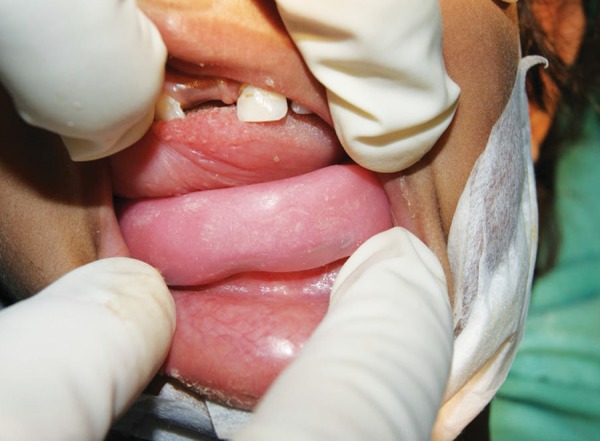
Occlusal splint in place

**Fig. 8:**
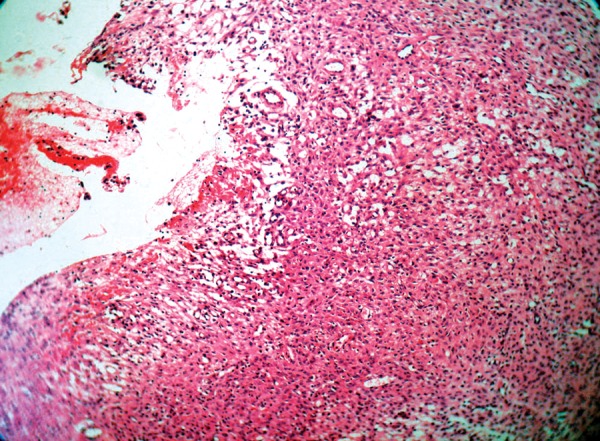
Photomicrograph of histologic picture (hematoxylin-eosin stain. Original magnification 10×)

A 3-year-old girl reported to the Department of Pedodontics and Preventive Dentistry, Modern Dental College and Research Center, Indore, Madhya Pradesh (India) with the chief complaint of painless swelling in the left lower third of the face since 2 months (Fig. 1). Past history revealed trauma (fall) in the same region when she was 1-year-old. Intraoral examination revealed asymptomatic, bony hard swelling in the buccal aspect of 73, 74, 75 (Fig. 2). Mandibular occlusal radiograph (Fig. 3) and orthopantomograph (Fig. 4) revealed a solitary well defined, unilocular radiolucency on left side of body of the mandible, crossing midline and extended to the mesial aspect of 83. Superiorly extended between the developing tooth follicles producing a scalloped appearance and showed thinning and intact inferior cortex. Surgical exploration with curettage was planned (Fig. 5). Prior to surgical exploration patients upper and lower arches impressions were made and working models were prepared. Acrylic splint (Fig. 6) was fabricated covering the full lower arch to be cemented using luting cement. The splint was planned^13,14^ to prevent fracture of the fragile mandibular walls after surgery. Surgical exploration of the lesion followed by curettage revealed, small bone chips with parts of the membrane and minimal amount of soft tissue which then were submitted for histopathological study. After inducing bleeding into the cavity it was surgically approximated by sutures. The custom made acrylic splint (Fig. 7) was cemented in place by luting cement. Histopathological report was suggestive of traumatic bone cyst (Fig. 8).

**Fig. 9:**
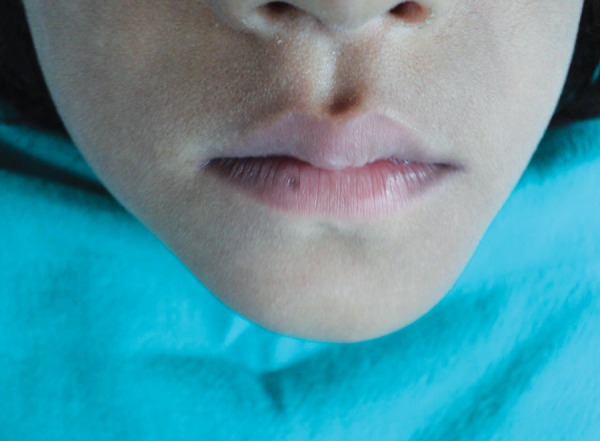
Postoperative extraoral photograph

**Fig. 10:**
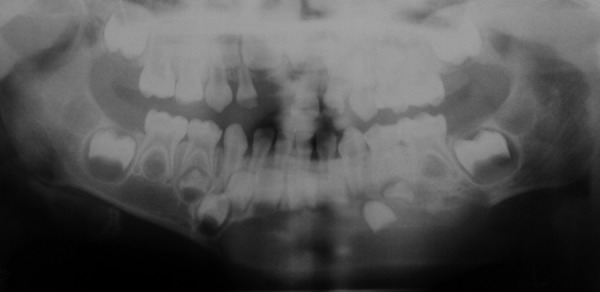
Postoperative panoramic radiograph showing bone formation at superoposterior aspect of 36 region

Patient was recalled periodically to access the bone formation. Clinical examination after 3 months reveled uneventful healing with regression of swelling and had normal mandibular contour (Fig. 9). Postoperative (3 months) panoramic radiograph showed bone formation at superopostero aspect at the periapex of 36 region without any fracture of the cortical plates of surgically curetted mandibular region (Fig. 10).

## DISCUSSION

The widely recommended treatment for TBC is surgical exploration followed by curettage of bony walls, which may provoke bleeding in the cavity, and hemorrhage forms clot in the cavity which is eventually replaced by bone and enhancing the healing process.^1,2,10,15,16^ It is believed that in some cases, especially younger patients there may be a spontaneous resolution.^1,10^ Few have even tried injection of autogenous blood, insertion of gel foam saturated with thrombin and penicillin, bone allografts or application of methyl prednisolone acetate to cavity.^17^

After surgical exploration with or without curettage, obliteration of the defect by new bone formation is generally rapid. Even large defects may show normal radiographic findings within 3 to 12 months after exploration.^1,16^

The multitude of the names applied to this lesion ^4^ attests to the lack of understanding of the true etiopathogenesis, several theories have been proposed.^10,11^

(1) Infection of bone marrow, (2) cystic degeneration of existing bone tumor, (3) changes and reduction in osteogenic activity, (4) ischemic necrosis of the fatty bone marrow, (5) low-grade chronic infection, (6) imbalance between the osteoclastic and osteoblastic activity due to trauma, (7) developmental defect, (8) failure of mesenchymal tissue to form bone and cartilage, and instead becomes immature as multiple bursa-like synovial cavities.^10^

The problems associated with immediate restoration of large mandibular defects are discussed as it holds key to the overall outcome of young patient’s rehabilitation. The pedodontist, working in close cooperation with the surgeon, may be called upon to construct an appliance that will serve after surgery has been performed on a patient. The design of the appliance, which may be intraoral or extraoral, will vary depending on the area involved, the type of operation performed and the limitations of the surgery. Knowledge of the patient condition, postoperative care is essential in order to keep the time required for patient rehabilitation to a minimum. Where practicable, splinting should be done immediately to avoid the common complications of fracture and occlusal disharmony.

## References

[1] Arsinoi AX, Konstantinos IC, Konstantinos T, Vasilios AP, Stavros IP (2006). Traumatic bone cyst of the mandible of possible iatrogenic origin: A case report and brief review of the literature.. Head and Face Medicine.

[2] Virchow R. (1875). About the formation of bone cysts. Meeting of the Academy of Sciences..

[3] Bhoosreddy AR, Gadgil RM, Bhoosreddy SA, Velankiwar GN (2010). Solitary bone cyst: A case report and review of literature.. J Ind Acad Oral Med Radiol.

[4] Saito Y, Hoshina Y, Nagamine T, Nakajima T, Suzuki M, Hayashi T, (First Department of Oral and Maxillofacial Surgery, School of Dentistry, Niigata University, Japan), (1992). Simple bone cyst. A clinical and histopathologic study of fifteen cases.. Oral Surg Oral Med Oral Pathol.

[5] Suei Y, Taguchi A, Tanimoto K, (Department of Oral and Maxillofacial Radiology, Hiroshima University Hospital, 1-2-3 Kasumi, Minami-ku, Hiroshima 734-8553, Japan. suei@hiroshima-u.ac.jp), (2007). A comparative study of simple bone cysts of the jaws and extracranial bones.. Dentomaxillofac Radiol.

[6] Hall AM, Orth D (1976). The solitary bone cyst. Report of two cases.. Oral Surg Oral Med Oral Pathol.

[7] White SC, Pharoah MJ (2009). Oral Radiology: Principles and Interpretation.

[8] Langlais RP., Langland OE. (1995). Diagnostic imaging of the jaws..

[9] Freedman GL, Beigleman MB (1985). The traumatic bone cyst: A new dimension.. Oral Surg Oral Med Oral Pathol.

[10] Perdigão PF, Silva EC, Sakurai E, Soares de Araújo N, Gomez RS, (Department of Oral Surgery and Pathology, School of Dentistry, Universidade Federal de Minas Gerais, Belo Horizonte, Brazil), (2003). Idiopathic bone cavity: A clinical, radiographic, and histological study.. Br J Oral Maxillofac Surg.

[11] Forssell K, Forssell H, Happonen RP, Neva M, (Institute of Dentistry, University of Turku, Finland), (1988). Simple bone cyst. Review of the literature and analysis of 23 cases.. Int J Oral Maxillofac Surg.

[12] Kuttenberger JJ, Farmand M, Stöss H, (Department of Oral and Maxillofacial Surgery, University of Erlangen-Nuremberg, Germany), (1992). Recurrence of a solitary bone cyst of the mandibular condyle in a bone graft. A case report.. Oral Surg Oral Med Oral Pathol.

[13] Williams JL (1973). Reconstruction of mandibular defects.. Int J Oral Surg.

[14] Beder OE (1948). Postsurgical prosthesis.. Oral Surg Oral Med Oral Pathol.

[15] Thoma KH (1995). A symposium on bone cysts (editorial).. Oral Surg.

[16] Pogrel MA (1987). A solitary bone cyst possibly caused by removal of an impacted third molar.. J Oral Maxillofac Surg.

[17] Huebner GR, Turlington EG (1971). So-called traumatic (hemorrhagic) bone cysts of the jaws. Review of the literature and report of two unusual cases.. Oral Surg Oral Med Oral Pathol.

